# Sex and N-terminal pro B-type natriuretic peptide: The potential mediating role of iron biomarkers

**DOI:** 10.3389/fcvm.2022.897148

**Published:** 2022-11-14

**Authors:** Farnaz Khatami, Taulant Muka, Dion Groothof, Martin H. de Borst, Chepkoech Buttia, Gaston van Hassel, Iris Baumgartner, Daan Kremer, Stephan J. L. Bakker, Arjola Bano, Michele F. Eisenga

**Affiliations:** ^1^Institute of Social and Preventive Medicine (ISPM), Graduate School of Health Sciences, University of Bern, Bern, Switzerland; ^2^Department of Community Medicine, Tehran University of Medical Sciences, Tehran, Iran; ^3^Epistudia, Bern, Switzerland; ^4^Division of Nephrology, Department of Internal Medicine, University of Groningen, Groningen, Netherlands; ^5^Department of Angiology, Swiss Cardiovascular Center, Inselspital, Bern University Hospital, University of Bern, Bern, Switzerland; ^6^Department of Cardiology, Bern University Hospital, University of Bern, Bern, Switzerland

**Keywords:** sex, NT-proBNP, iron biomarkers, iron status, cardiac markers, natriuretic peptides, general population

## Abstract

**Background:**

Levels of N-terminal pro B-type natriuretic peptide (NT-proBNP), a marker of heart failure and cardiovascular risk, are generally higher in women than men. We explored whether iron biomarkers mediate sex differences in NT-proBNP levels.

**Methods:**

We included 5,343 community-dwelling individuals from the Prevention of Renal and Vascular Endstage Disease study. With linear regression analyses, we investigated the association of sex and iron biomarkers with NT-proBNP levels, independent of adjustment for potential confounders. The assessed iron biomarkers included ferritin, transferrin saturation (TSAT), hepcidin, and soluble transferrin receptor (sTfR). Next, we performed mediation analyses to investigate to which extent iron biomarkers influence the association between sex and NT-proBNP.

**Results:**

Of the included 5,343 participants, the mean standard deviation age was 52.2 ± 11.6 years and 52% were females. After adjustment for potential confounders, women compared to men, had higher NT-proBNP (β = 0.31; 95%CI = 0.29, 0.34), but lower ferritin (β = –0.37; 95%CI = –0.39, –0.35), hepcidin (β = –0.22, 95%CI = –0.24, –0.20), and TSAT (β = –0.07, 95% CI = –0.08, –0.06). Lower ferritin (β = –0.05, 95%CI = –0.08, –0.02), lower hepcidin (β = –0.04, 95%CI = –0.07, –0.006), and higher TSAT (β = 0.07; 95%CI = 0.01, 0.13) were associated with higher NT-proBNP. In mediation analyses, ferritin and hepcidin explained 6.5 and 3.1% of the association between sex and NT-proBNP, respectively, while TSAT minimally suppressed (1.9%) this association.

**Conclusion:**

Our findings suggest that iron biomarkers marginally explain sex differences in levels of NT-proBNP. Future studies are needed to explore causality and potential mechanisms underlying these pathways.

## Introduction

Cardiovascular disease (CVD) is the major cause of mortality in both women and men. However, the manifestations of CVD differ by sex and the pathophysiological mechanisms underlying these sex-based differences remain incompletely understood (1, 2). Circulating biomarkers may provide additional insight into these differences. N-terminal pro-brain natriuretic peptide (NT-proBNP) levels are secreted from cardiomyocytes in response to atrial or ventricular wall stretch (3, 4). NT-proBNP levels are strong predictors of cardiovascular events and mortality beyond traditional risk factors in the general population. In addition, NT-proBNP levels are important guides in the clinical management of patients with heart failure (HF) and asymptomatic left ventricular dysfunction (5, 6). Interestingly, women generally have higher circulating NT-proBNP levels than men (2, 7–9).

While the factors contributing to sex differences in NT-proBNP are not well understood, iron metabolism could play a role. Similar to NT-proBNP, levels of iron biomarkers differ between men and women. Iron status reflected by ferritin and transferrin saturation (TSAT) is generally lower in women of reproductive age due to monthly blood loss from menstruation. After menopause, iron will no longer be excreted and concentrations of circulating ferritin tend to increase, although women may not reach the levels of body iron stores that are found in men (10–12). Levels of hepcidin, a liver-derived peptide orchestrating systemic iron homeostasis, have been reported to be lower in healthy women than in men (13). In addition, there is evidence linking iron metabolism to NT-proBNP and HF pathophysiology. Recent studies in acute as well as chronic HF have linked lower levels of serum hepcidin and elevated soluble transferrin receptor (sTfR), a marker of both functional iron deficiency and erythroid activity, with higher levels of NT-proBNP (14–16). In a clinical trial of anemic patients with chronic HF and kidney failure, intravenous iron therapy without recombinant human erythropoietin substantially reduced NT-proBNP levels (17). In addition, data from the general population have shown that higher serum ferritin levels are associated with increased risk of atrial fibrillation, and new-onset HF, with some studies suggesting sex dependency of this association (11, 18, 19). Iron metabolism is associated with cardiomyopathy, atrial fibrillation and systolic and diastolic cardiac function, which may consequently affect NT-proBNP levels (15, 20–22). We therefore hypothesized that differences in iron parameters and changes in iron status may partly explain the sex differences observed in the levels of NT-proBNP (23). To test this hypothesis, we aimed to: (i) investigate the association of sex with NT-proBNP; (ii) investigate the associations of sex with iron biomarkers and iron biomarkers with NT-proBNP; and (iii) assess the potential mediating role of iron biomarkers in the association between sex and NT-proBNP.

## Materials and methods

### Study design

We performed this study using data obtained from Prevention of REnal and Vascular ENd-stage Disease (PREVEND), a large prospective, well-characterized observational cohort study. This study was designed to prospectively conduct an investigation into the natural process of increased levels of urinary albumin excretion and its association to renal outcomes and CVD in the general population. Details of the study protocol have been described elsewhere (24). Briefly, in the period 1997–1998, all inhabitants of the city of Groningen, the Netherlands, aged 28–75 years (*n* = 85,421) received a vial to collect an early morning urine sample, and a questionnaire on demographics, disease history, smoking habits, and use of medication. The response rate was 47.8% (40,856 subjects). After the exclusion of pregnant women and insulin-dependent diabetes mellitus patients, subjects with a urinary albumin excretion ≥ 10 mg/L (*n* = 6,000) and a randomly selected control group with a urinary albumin excretion < 10 mg/L (*n* = 2,592) completed the study protocol and formed the baseline PREVEND cohort population (*n* = 8,592). In this cohort, CVD patients and participants with hypercholesterolemia and hypertension were included. For the present analyses, we used data from the second survey, which took place between 2001 and 2003 (*n* = 6,894), since only for this visit, NT-proBNP and iron biomarkers were available. We excluded patients with heart failure (*n* = 224), those with left ventricular hypertrophy (*n* = 189), CVD patients (*n* = 336); iron supplement users (*n* = 31); those who had CRP > 10 mg/l (*n* = 173); those with missing NT-proBNP (*n* = 257); and those without iron biomarkers information (*n* = 341) (Supplementary Figure 1). Thus, there were 5,343 participants included in the final analysis.

The PREVEND study protocol was approved by the medical ethical committee of the University Medical Center Groningen (UMCG; ethical approval number MEC 96/01/022). All subjects provided written informed consent.

### Iron parameters and N-terminal pro B-type natriuretic peptide

Serum iron (μmol/L) was measured by colorimetric assay, ferritin (μg/L) by immunoassay, and transferrin (g/L) by immunoturbidimetric assay (all Roche Diagnostics, Mannheim, Germany). TSAT (%) was determined as [serum iron ÷ (25 × transferrin)] × 100. Serum hepcidin (nmol/L) was calculated with a competitive enzyme-linked immunosorbent assay with intra- and interassay coefficients of variations of 8.6 and 16.2%, respectively. An automated homogenous immunoturbidimetric assay with intra and interassay coefficients of variations < 2% and < 5% quantified sTfR (25). N-terminal pro-BNP measurements were performed on Elecsys 2010 analyzer, a commercially available electrochemiluminescent sandwich immunoassay (Elecsys proBNP, Roche Diagnostics, Mannheim, Germany) (26). All hematologic measurements were measured in fresh venous blood. Aliquots of these samples were stored immediately at -80°C until further analysis (25).

### Measurements of covariates

Blood pressure was calculated based on the mean of the two measurements. Body mass index (BMI) was defined as weight in kilograms divided by the square of height in meters. Type 2 diabetes mellitus was considered as a fasting glucose level of ≥ 7.0 mmol/L (126 mg/dL), a non-fasting glucose level of ≥ 11.1 mmol/L (200 mg/dL), or self-reported use of antidiabetic drugs. Estimated glomerular filtration rate (eGFR) predicted by the Chronic Kidney Disease Epidemiology Collaboration equation (27). Smoking was defined as none, former or current smokers. Alcohol was categorized to none, low, moderate, or high users. Hemoglobin (g/dL) was measured using a Coulter Counter STKS sum (Coulter Corporation, Miami, FL, USA). Concentrations of total cholesterol were measured with standard methods. High-sensitivity C-reactive protein (hs-CRP) was measured using nephelometry with a threshold of 0.175 mg/L and intra- and interassay coefficients of variations of < 4.4 and 5.7%, respectively. Using of anti-lipid and antihypertensive medications was self-reported (18).

### Statistical analyses

Baseline characteristics are expressed as mean, standard deviation (SD), median (interquartile range), or number (percentage) for normally distributed, skewed, and categorical data, respectively. Sex-stratified differences in baseline characteristics of participants were assessed with independent-samples *t*-test or Mann-Whitney *U*-test for continuous variables, and the chi-squared test for categorical variables. For further analyses, skewed variables, including NT-proBNP, ferritin, hepcidin, sTfR, TSAT, BMI, total cholesterol, hs-CRP, eGFR, SBP, and hemoglobin were natural log-transformed to achieve a normal distribution. Multiple linear regression models were used to investigate the association of sex with iron biomarkers (ferritin, hepcidin, TSAT, and sTfR), as well as sex and iron biomarkers with NT-proBNP. All analyses were adjusted for age (model 1) and additionally for BMI, alcohol use, and smoking (model 2). Model 2 is based on the potential confounders identified in the literature and was the main model we used in mediation analysis. Missing observations in covariates were handled using multiple imputation procedures (*n* = five imputations). Pooled regression coefficients (β) and 95% Confidence Intervals were generated using Rubin’s method (28). The percentage of missing values in covariates was lower than 3% (ranging from 0.1 to 14%), except for CRP for which there were 14% of missing values. Potential multicollinearity was excluded by assessment of variance inflation factors. Interactions with age were explored for all analyses in model 2 by adding an interaction term for the interaction between exposure of interest and age to the second model and tested. Lastly, to understand the extent to which sex differences in NT-proBNP levels are mediated by levels of one or more iron biomarkers, mediation analyses were employed (Supplementary Figure 2) (29, 30). Only the iron biomarkers that were both associated with sex and NT-proBNP were evaluated. We tested the following paths: (i) direct effect of the exposure on the outcome; (ii) indirect effect of the exposure, *via* a mediator on the outcome; (iii) sum of direct effect and indirect effect as total effect; and (iv) the mediated proportion (indirect effect/total effect). To perform mediation analysis, the relationship between exposure to the mediator variables and the association between the mediator variables and the outcome must be statistically significant. The threshold for statistical significance in all probabilities was a two-tailed *P*-value < 0.05. Statistical analyses were performed using IBM SPSS software, version 27.0, PROCESS v3.5 by Preacher and Hayes (31).

### Sensitivity analyses

To examine the robustness of our results, we performed several sensitivity analyses. To account for the potential influence of mediators in the association of sex and iron biomarkers with NT-proBNP, as well as of sex with iron biomarkers, we additionally adjusted our analyses for total cholesterol, use of anti-lipid and antihypertensive drugs, hs-CRP, eGFR, systolic blood pressure, and type 2 diabetes (model 3) and (ii) additionally for hemoglobin (model 4). Further, we explored whether iron status as a categorical variable was explaining sex differences in NT-proBNP levels. Participants were classified into three groups in terms of iron status; iron deficiency (ID) as a ferritin level < 30 μg/L for men and < 15 μg/L for women; iron overload as fourth quartiles of TSAT (30.5%) and ferritin (168 μg/L) and the rest of the subjects as normal group (25, 32, 33). In addition, we analyzed a spectrum of definitions with ID as ferritin < 30 μg/L for both sex; the low iron as first quartiles of TSAT and ferritin; and the TSAT > 45% as iron overload (33–35). In addition, we conducted a similar analysis in the main model with the exclusion of diabetic participants and those undergoing anti-hypertensive or lipid-lowering drugs. Finally, to investigate the effect of age, we divided the participants into two groups based on the median age, 51 years, and repeated the main model.

## Results

Table 1 displays baseline characteristics of the 5,343 eligible participants, stratified by sex. Mean age was 52.2 ± 11.6 years, and 52% were women. Median NT-proBNP level was 38 (20–71) ng/L and was higher in women than in men (51 vs. 25 ng/L, *p* < 0.001). Women had a lower BMI, lower systolic blood pressure (SBP), and less type 2 diabetes. Compared with men, women had a lower TSAT, ferritin and hepcidin concentrations (*p* < 0.001 for each). Iron status was significantly different in men and women (*p* < 0.001), with absolute iron deficiency (AID) being more prevalent in women.

**TABLE 1:** Baseline characteristics of participants by sex.

Characteristics	Total (5,343)	Female (2,805)	Male (2,538)	*P-value* *
**Demographic**				
Age (years)	52.2 ± 11.6	51.9 ± 11.2	52.5 ± 11.9	0.04
**Clinical**				
Body-mass index, kg/m2	26 (23.6–28.8)	25.5 (23–28.8)	26.3 (24.1– 28.7)	<0.001
Systolic blood pressure, mmHg	122 (112–134)	117 (108– 130)	127 (117– 138)	<0.001
Prevalent type 2 diabetes mellitus (*n*, %)	264 (5)	123 (4.4)	141 (5.6)	0.05
**Alcoholic behavior**				
No	1,269 (24)	850 (30.5)	419 (16.7)	<0.001
1–4 units/mo (*n*, %)	916 (17.3)	548 (19.7)	368 (14.7)	
2–7 units/wk (*n*, %)	1,716 (32.4)	835 (30)	881 (35.1)	
1–3 units/d (*n*, %)	1,170 (22.1)	493 (17.7)	677 (27)	
≥ 4 units/d (*n*, %)	225 (4.2)	59 (2.1)	166 (6.6)	
**Smoking behavior**				
Never	1,625 (30.8)	931 (33.2)	694 (27.3)	<0.001
Former	2,182 (41.3)	1,066 (38)	1,116 (44)	
Current	1,473 (27.9)	778 (27.7)	695 (27.4)	
Use of antihypertensive drugs (*n*, %)	885 (16.6)	467 (16.7)	418 (16.5)	0.88
Use of lipid-lowering drugs (*n*, %)	341 (6.4)	181 (6.5)	160 (6.3)	0.83
**Laboratory**				
Plasma NT-proBNP, ng/L	38 (20–71)	51 (30.5–85)	25 (13–47.3)	<0.001
Total cholesterol, mmol/l	5.4 (4.7–6.1)	5.4 (4.7– 6.1)	5.4 (4.8– 6.1)	0.21
eGFR, ml/min per 1.73 m2	92.9 (79.4–108.2)	106.2 (94– 115.4)	81.1 (70.5– 90.5)	<0.001
High-sensitivity C-reactive protein, mg/l	1.2 (0.6–2.6)	1.3 (0.6– 2.9)	1.1 (0.6– 2.4)	<0.001
TSAT (%)	24.3 (19.1–30.5)	23.1 (17.5– 28.8)	26.1 (21–32.4)	<0.001
Serum ferritin, μg/L	94 (46–168)	58 (29– 108)	143 (86– 226)	<0.001
Serum hepcidin, nmol/l	3 (1.6–4.8)	2.3 (1.1 –4)	3.8 (2.4– 5.5)	<0.001
Plasma sTfR, mg/l	2.4 (2.1–2.9)	2.4 (2– 3)	2.5 (2.1– 2.9)	0.13
Hemoglobin, g/dl	13.7 (12.9–14.5)	13 (12.4–13.5)	14.5 (13.9–15.1)	<0.001
**Iron status**				
Absolute iron deficiency	379 (7.1)	288 (10.3)	91 (3.6)	<0.001
Normal group	4,482 (83.9)	2,425 (86.5)	2,057 (81)	
Iron overload	482 (9)	92 (3.3)	390 (15.4)	

Data are presented as mean ± *SD* or median (interquartile range) for continuous variables and number (%) for categorical variables.

NT-proBNP, indicates N-terminal pro B-type natriuretic peptide; eGFR, estimated glomerular filtration rate; TSAT, transferrin saturation; and sTfR, soluble transferrin receptor.

*Comparison between sex with independent-samples *T*-Test, Mann-Whitney *U* test, and chi-squared test.

### Sex, iron biomarkers, and N-terminal pro B-type natriuretic peptide

Table 2 presents the association of sex and iron biomarkers with NT-proBNP. In model 1 (adjusted for age) and model 2 (adjusted for age, BMI, smoking, and alcohol), women had higher levels of NT-proBNP than men did (β = 0.31, 95% CI: 0.29, 0.34). After adjustment for sex and potential confounders in model 2, lower ferritin (β = –0.05, 95% CI: –0.08, –0.02), lower hepcidin (β = –0.04, 95% CI: –0.07, –0.006) and higher TSAT (β = 0.07, 95% CI: 0.01, 0.13) levels were associated with higher levels of NT-proBNP. Table 3 shows the association of sex with iron biomarkers. In model 2, women had lower levels of ferritin (β = –0.37, 95% CI: –0.39, –0.35), hepcidin (β = –0.22, 95% CI: –0.24, –0.20) and TSAT (β = –0.07, 95% CI: –0.08, –0.06) compared to men. The association between sex and iron biomarkers in model 1 was similar to model 2. No evidence of significant interaction of iron biomarkers and sex with age was found (all *p*-values < 0.05).

**TABLE 2:** Association of sex and iron biomarkers with NT-proBNP.

	Model 1*	Model 2^†^
		
	Beta (95% CI)^§^	Beta (95% CI)
Sex (ref = m)	0.32 (0.30, 0.34)	0.31 (0.29, 0.34)
Ferritin, μg/L^‡^	–0.07 (–0.10, –0.04)	–0.05 (–0.08, –0.02)
Hepcidin, nmol/l	–0.05 (–0.08, –0.02)	–0.04 (–0.07, –0.006)
sTfR, mg/l	–0.09 (–0.16, –0.02)	–0.04 (–0.11, 0.03)
TSAT, %	0.10 (0.04, 0.16)	0.07 (0.01, 0.13)

CI, indicates confidence interval; sTfR, soluble transferrin receptor; TSAT, transferrin saturation; and BMI, body mass index.

*Model 1 was adjusted for age.

^†^Model 2 was adjusted for age, BMI, smoking, and alcohol use.

^‡^Analyses of iron biomarker were additionally adjusted for sex.

^§^Statistical test: multiple linear regression. All iron biomarkers and NT-proBNP were log transformed.

**TABLE 3:** Association of sex with iron biomarkers.

	Model 1*	Model 2^†^
		
	Beta (95% CI)^‡^	Beta (95% CI)
Ferritin, μg/L	–0.40 (–0.42, –0.38)	–0.37 (–0.39, –0.35)
Hepcidin, nmol/l	–0.23 (–0.25, –0.21)	–0.22 (–0.24, –0.20)
sTfR, mg/l	0.00 (–0.01, 0.01)	–0.01 (–0.01, 0.003)
TSAT, %	–0.07 (–0.08, –0.06)	–0.07 (–0.08, –0.06)

CI, indicates confidence interval; sTfR, soluble transferrin receptor; TSAT, transferrin saturation; and BMI, body mass index.

*Model 1 was adjusted for age.

^†^Model 2 was adjusted for age, BMI, smoking, and alcohol use.

^‡^Statistical test: multiple linear regression. All iron biomarkers were log transformed. Reference category for sex is men.

### Mediation analyses and role of iron biomarkers

Since ferritin, hepcidin, and TSAT differed by sex and were associated with NT-proBNP levels, we subsequently tested the potential mediating role of these iron parameters. In model 2 as the main model, ferritin, hepcidin, and TSAT were mediators of the association between sex and NT-proBNP. Indeed, 6.5 and 3.1% of the association between sex and NT-proBNP was explained by ferritin and hepcidin, respectively. TSAT minimally suppressed (1.9%) the association of sex and NT-proBNP (negative effect) (Table 4).

**TABLE 4:** Mediation analyses of iron biomarkers on the association between sex and NT-proBNP.

	Model 1*			Model 2^†^		
		
Iron biomarkers	Effects^#^	Coefficient (95% CI)	Mediated proportion (%)^‡^	Effects	Coefficient (95% CI)	Mediated proportion (%)^‡^
Ferritin, μg/L	Indirect Total	0.03 (0.02, 0.04) 0.32 (0.30, 0.34)	9.4%^§^	Indirect Total	0.02 (0.01, 0.03) 0.32 (0.30, 0.34)	6.5%^§^
Hepcidin, nmol/l	Indirect Total	0.02 (0.01, 0.02) 0.32 (0.30, 0.35)	6.5%^§^	Indirect Total	0.01 (0.002, 0.02) 0.32 (0.30, 0.34)	3.1%^§^
TSAT, %	Indirect Total	–0.01 (–0.01, –0.004) 0.32 (0.30, 0.34)	3.1%^||^	Indirect Total	–0.006 (–0.01, –0.001) 0.31 (0.30, 0.34)	1.9%^||^

CI, indicates confidence interval; TSAT, transferrin saturation; and BMI, body mass index. In mediation analysis, only the iron biomarkers that were both associated with sex and NT-proBNP were evaluated.

*Model 1 was adjusted for age.

^†^Model 2 was adjusted for age, BMI, smoking, and alcohol use.

^‡^Mediated proportion is calculated as the ratio of indirect effect to the total effect and is expressed in percentage.

^§^Positive mediator effect. ^||^Negative mediator effect.

^#^Statistical test: mediation analysis. All iron biomarkers and NT-proBNP were log transformed.

### Sensitivity analyses

The results on the association of sex and iron biomarkers with NT-proBNP did not materially change after adjustment for all confounding and potential mediating factors (model 3); however, the association between hepcidin and NT-proBNP was abolished, while the association of sTfR became statistically significant (Supplementary Table 1). Further adjustment for hemoglobin levels (model 4) did not greatly change the association between sex and NT-proBNP but abolished the association between ferritin and NT-proBNP (Supplementary Table 1). The effect of sex on iron biomarkers in fully adjusted model 3 was similar to the main model, except women had higher levels of sTfR. However, subsequent adjustment for hemoglobin levels attenuated the association, such that TSAT and sTfR were no longer significantly associated with sex (Supplementary Table 2).

In mediation analysis in model 3, ferritin explained 4% of the association between sex and NT-proBNP (positive effect), while TSAT (suppressor effect) explained 2% (Supplementary Table 3). Mediation analysis in model 4 could not be performed due to no compliance to model assumptions (see section “Materials and methods). In model 2, using body iron status as a categorical variable showed (i) no association between body iron status and NT-proBNP; (ii) women having higher odds than men for being with AID; (iii) and no mediation analysis of iron status in the association between sex and NT-proBNP, due to no compliance to model assumptions. Additional information about the associations of iron status with NT-proBNP and sex is provided in Supplementary Tables 4, 5. The results did not materially change when other definitions of AID and iron overload were used. Lastly, the analyses excluding participants who reported use of anti-hypertensive medications, lipid-lowering drugs, or having diabetes (*n* = 1,490) showed similar findings to the main analyses (Supplementary Tables 6–8). In the investigation stratified by median age, the association of iron biomarkers was consistent but non-significant. In the mediation analysis, only the suppressor role of TSAT was retained (Supplementary Tables 9–11).

## Discussion

This is the first study to explore the potential mediating effect of iron biomarkers on the association between sex and NT-proBNP. We showed that NT-proBNP levels differ by sex, with women having higher levels compared to men; this difference was independent of potential confounding factors. Levels of ferritin, hepcidin, and TSAT also differed between men and women, and could mediate up to 8% of the association between sex and NT-proBNP.

### Association between sex and N-terminal pro B-type natriuretic peptide

NT-proBNP is broadly used as a biomarker of HF and asymptomatic left ventricular dysfunction. Compared to men, women have higher NT-proBNP levels, regardless of their hormonal and menopausal status (36–39). A study in 746 participants from the general population reported that female sex was the strongest independent predictor of higher NT-proBNP (39). The Japan Morning SurgeHome Blood Pressure (J-HOP) Study reported a mean difference of NT-proBNP of approximately 10 pg/mL between women and men (54.7 vs. 44.9 pg/mL, *p* < 0.001) (7). Conversely, other studies have also highlighted the impact of menopause on the association between sex and NT-proBNP; (8, 40) for example, a study in 3,439 men and women found that NT-proBNP is higher in premenopausal women but closer to men in postmenopausal women (1). The biological basis of sex differences in NT-proBNP has not yet been elucidated. While sex hormones have been suggested to explain these differences, our study supports the hypothesis that body iron status could also play a mediating role (41). One of the important factors that can influence the association between sex and NT-proBNP is menopausal status. We did not have information on the menopausal status of women included in our analysis and could not provide an analysis of menopausal status. However, the median age of women included in our study was 51 years old, which is similar to the median age of 51.4 years in high-income countries (42). Suggesting that the majority of women included in our study were premenopausal women, in the menopause transition, or a few years from the start of menopause, we have included both premenopausal and postmenopausal in our analysis. In our study, assessing participants according to the median age could not accurately examine the effect of age or menopause onset and will need to be delineated in more detail in the future. Similarly, previous studies have also shown that androgens but not estrogens are associated with levels of NT-proBNP (41), which should be further explored in future studies. Cao and et al. showed in a community-based population study in Beijing that resting heart rate was positively associated with plasma NT-proBNP in elderly, independent of sex. On the other hand, they found increased levels of NT-proBNP in females. Therefore, it is currently not well understood whether increased heart rate in women is a factor in increasing NT-proBNP levels (43).

### Association between sex and iron biomarkers

Our study showed that women have lower levels of ferritin, hepcidin, and TSAT compared to men. The lower levels of ferritin in women could be partly explained by the loss of iron during menstruation. Although iron begins to accumulate during the end of the menstrual period, it takes years to become similar to levels observed in men (13). The third National Health and Nutrition Examination Survey conducted in 20,040 US individuals > 17 years of age from the general population, showed that levels of ferritin in women started increasing by the age of 40–49 years old, and only after age of 90 years old would achieve similar values to men (44). In addition, new evidence shows a direct link between estrogen and systemic iron metabolism. Women, especially at young age, have higher levels of estrogen than men (45). Treatment with estrogens (E2) can decrease hepcidin mRNA expression, which can explain our findings of women having lower levels of hepcidin (46). Hepcidin is the master regulator of the iron homeostasis. Hepcidin regulates iron homeostasis by binding directly and causing internalization and degradation of ferroportin, the sole known iron exporter. As such, hepcidin prevents iron excess by inhibiting the absorption of iron and promoting its sequestration in macrophages, monocytes, and hepatocytes. Thus, hepcidin restricts the occurrence of transferrin-unbound iron and consequently the production of reactive oxygen species (ROS). Studies have shown that increased iron saturation and inflammation stimulate hepcidin expression, while increased erythropoiesis and iron deficiency reduce hepcidin construction and raise access to iron ions in the blood. In conditions of decreased iron levels, such as in premenopausal women and the chronic HF populations, the production of hepcidin is down-regulated, allowing more iron to enter into the blood, which further binds transferrin (14, 47). Serum hepcidin and ferritin concentrations are strongly correlated in healthy subjects (*r* = 0.63, *P* < 0.001) [10]. This correlation is also observed in HF patients with ID, in whom lower hepcidin expression is associated with low serum ferritin (depleted iron stores), and negative tissue iron balance (high serum sTfR and low TSAT) (15, 16).

### Association of iron biomarkers with N-terminal pro B-type natriuretic peptide and heart failure

To our knowledge, this is the first comprehensive study to investigate the association of different iron biomarkers with NT-proBNP levels in the general population. The evidence on the association between iron biomarkers and NT-proBNP levels is limited and mainly derived from studies conducted in patients with HF. Jankowska and colleagues demonstrated that 37% of HF patients with concomitance of low hepcidin and high sTfR (the most profound ID) had high NT-proBNP and low hemoglobin (*P* < 0.05) concentrations (15). In a double-blind, randomized, placebo-controlled study, intravenous iron therapy of 5-week for 40 HF patients with ID had a significant improvement not only in ferritin, TSAT, hemoglobin levels but also decreased NT-proBNP levels, compared to the control group (17). Increasing NT-proBNP predicts onset of HF in the general population, as well as prognosis of HF patients (5, 6). Previous epidemiological studies in the general population have shown an association between both low and high body iron stores and risk for HF, although the findings are inconclusive and can differ by sex.

The Atherosclerosis Risk in Communities study including 1,063 non-heart failure participants with a mean follow-up of 21 ± 4.6 years found that subjects with low ferritin (< 30 ng/mL) (HR: 2.24, CI 95%: 1.15–4.35), as well high ferritin (> 358 ng/mL) (HR: 1.81, CI 95%: 1.04–3.25) had the highest rate of incident HF. In the same study, low ferritin levels were associated with incident HF after excluding participants with anemia, suggesting that the association of iron deficiency with incident HF is independent of anemia (19). In our study population (PREVEND), higher ferritin and hepcidin levels have been associated with increased risk for new-onset HF only in women, independent of established conventional cardiovascular risk factors (18). The meta-analysis of 35,799 individuals from three studies of the Danish general population showed increased ferritin concentration to be associated with increased risk of atrial fibrillation in the general population. While no association was found between ferritin concentrations and HF (11). It should be noted that these studies used only one single measure of iron biomarkers, which could have attenuated the observed effects considering iron biomarkers differ over time, especially in younger women (44). It can be assumed that elevated ferritin and hepcidin levels might be a reaction to low-grade inflammation to another pathophysiological process, which can further promote the development of HF (18). Additionally, higher circulating hepcidin and ferritin may indicate the early stage of HF (16). Some pathophysiological mechanisms might explain the association between low ferritin and incident HF. In a state of ID, there is impairment in cellular energetics due to a reduction in the activity of muscular oxidative enzymes and respiratory proteins, with structural alterations (e.g., mitochondrial swelling) and irregularities in sarcomere organization (48). ID causes mitotic arrest and apoptosis, altering the myocardial composition. Further, it might come up to cardiac hypertrophy through increasing catecholamines levels and finally contribute to the development of HF (19).

We observed a positive linear association of TSAT with NT-proBNP, independent of sex. While there is lack of data on the association between TSAT and NT-proBNP, in the general population, both low and high TSAT are significantly and independently associated with increased cardiovascular and all-cause mortality (49). It is worth mentioning that, low TSAT levels can reflect not only ID but also inflammatory (iron and transferrin as negative acute-phase reactant) and nutritional conditions (artificial elevation in malnutrition); therefore, despite its valuable clinical utility an accurate interpretation of the body’s iron status may be affected by these fluctuations (49). As well, Stack et al. manifested a strong independent j-shaped association of TSAT with all-cause and cardiovascular mortality in older men and younger women, independent of comorbid conditions, nutritional status, and socioeconomic indicators (50). The possible mechanism of this j-shaped association is a chronic ID that causes structural alterations in cardiomyocytes and impairs cardiac performance. On the other hand, studies showed that unbound iron becomes the initiating factor for the production of ROS, which results in tissue damage and loss of their function, especially in heart diseases (50). Unfortunately, we did not have markers of oxidative stress for this study and could not explore this pathway and additional studies are needed.

### Mediation analysis

**FIGURE 1:**
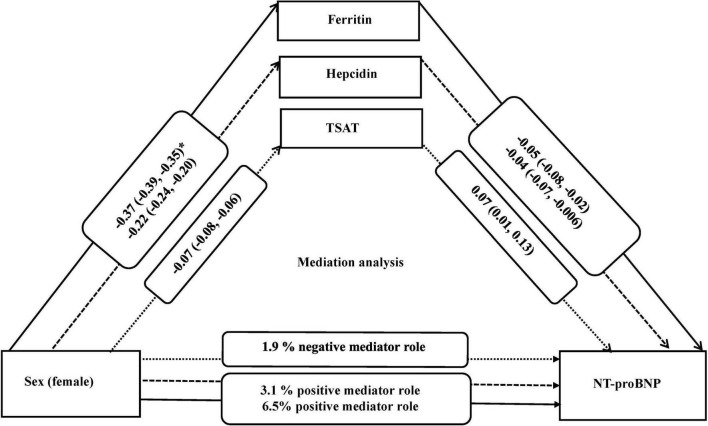
Mediation analysis for association of sex with NT-proBNP and the role of iron biomarkers, adjusted for (covariates) age, BMI, smoking, and alcohol use. *Beta (95% confidence interval). All iron biomarkers were log transformed. NT-proBNP indicates N-terminal pro B-type natriuretic peptide; TSAT, Transferrin saturation; and BMI, body mass index.

Our study hypothesized that sex impacts the levels of iron biomarkers, which can further influence the levels of NT-proBNP. Our mediation analysis showed that ferritin, hepcidin, and TSAT mediate up to 8% of the association between sex and NT-proBNP. This proportion of mediation is meaningful, given the multiple mechanisms underlying sex differences in NT-proBNP levels and cardiovascular risk. Compared to men, women had lower levels of ferritin and hepcidin, and in turn lower levels of ferritin and hepcidin were related to higher levels of NT-proBNP. These results suggest that the association of female sex with higher levels of NT-proBNP could be explained by the reduced levels of ferritin and hepcidin. On the other hand, female sex was linked to lower TSAT, and in turn lower TSAT was associated with lower levels of NT-proBNP. This suggests that TSAT may have a suppressive effect on the association between female sex and NT-proBNP (Figure 1). The exact underlying mechanisms that can explain the potential mediating role of specific iron parameters on the association between sex and NT-proBNP remain unclear. Hypothetically, hemoglobin levels could be involved in these pathways. Low hemoglobin levels and anemia can be one of the mechanisms linking iron deficiency to HF (19). In patients with CVD, an independent predictive value of hemoglobin was shown for BNP levels (51, 52). Furthermore, anemic patients with decreased concentration can also have elevated serum concentrations of NT-proBNP, even in the absence of chronic HF and regardless of sex (17). Our study indicated that adjustment for hemoglobin levels attenuated the association between iron biomarkers and NT-proBNP and that AID, independent of anemia, was associated with NT-proBNP levels. Further investigations are warranted to confirm these findings.

### Strengths and limitations

The major strengths of our study include its large sample size, as well as the assessment of a large number of biomarkers such as iron parameters. To our knowledge, our study is the first to investigate the role of iron biomarkers on the association between sex and NT-proBNP in the general population. The exclusion of participants with known CVD reduced the possibility of confounding by CVD. Several limitations warrant mentioning. Our study is cross-sectional, and thus causality cannot be established. Due to the observational design of the study, residual confounding might have biased the results. For example, ferritin and hepcidin as acute-phase reactants and transferrin as negative acute-phase reactant can be influenced by various inflammatory markers. It is thus possible that, despite adjustment for hs-CRP, inflammation has still influenced the observed associations. In addition, participants of our study lived in a European country, which calls for prudence when extrapolating these results to other populations. Lastly, we included both premenopausal and postmenopausal women in our analysis. Due to a lack of information on menopause status, we were unable to examine the role of menopause, which should be explored in future studies.

### Implications

The potential implications of identifying new biomarkers, such as iron biomarkers, that partially mediate some of the effect of sex on NT-proBNP, could help in gaining a more profound understanding of the pathophysiology of sex differences in cardiovascular health and identifying novel sex-specific therapeutic targets. However, future studies are needed to replicate our findings, and establish causality. If our hypothesis stands, future preventive strategies could emphasize the public health importance of monitoring iron biomarkers, and introducing preventive measures, such as increasing iron intake in women population to improve iron status. In addition, while the absolute differences we find in the association between iron biomarkers and NT-proBNP were not large enough to potentially have clinical implications, our results could have an impact at the population level. As the prevention paradox highlights, shifting the levels of a risk factor even in small units could be translated into large benefits at the population level.

## Conclusion

This study shows that the association between sex and NT-proBNP is partly mediated by levels of ferritin, hepcidin, and TSAT. Future large studies and clinical trials are needed to assess the causality of these findings, and to explore the implications that this mediating effect could have for prevention, diagnosis and treatment of preclinical cardiac disease of women and men.

## Data availability statement

The dataset used for the current study are available from the corresponding authors on reasonable request.

## Ethics statement

The PREVEND study protocol was approved by the Medical Ethical Committee of the University Medical Center Groningen (UMCG; ethical approval number MEC 96/01/022). The patients/participants provided their written informed consent to participate in this study.

## Author contributions

FK, TM, AB, and ME designed, conducted the study, and drafted the manuscript. FK, ME, MB, and GH involved in data collection, data cleaning, and analysis. FK, TM, AB, DG, MB, GH, DK, SB, IB, and CB critically interpreted the data. All authors reviewed the results and approved the final version of the manuscript.

## Abbreviations

AID: absolute iron deficiency
CVD: cardiovascular diseases
eGFR: estimated glomerular filtration rate
HF: heart failure
hs-CRP: high-sensitivity c-reactive protein
NT-proBNP: N-terminal pro B-type natriuretic peptide
PREVEND: Prevention of REnal and Vascular ENd-stage Disease
sTfR: soluble transferrin receptor
SBP: systolic blood pressure
TSAT: transferrin saturation.
