# Supplementary material for: Sex and N-terminal pro B-type natriuretic peptide: The potential mediating role of iron biomarkers
Source: Front Cardiovasc Med. 2022 Nov 14;9:897148. doi: 10.3389/fcvm.2022.897148 (PMC9703058; doi:10.3389/fcvm.2022.897148)
Supplement: Supplementary file 1 [file Data_Sheet_1.docx]

Supplementary Table 1. Association of sex and iron biomarkers with NT-proBNP, in model 3 and 4

|  | Model 3^*^ | Model 4^†^ |
| --- | --- | --- |
|  | Beta (95% CI) ^§^ | Beta (95% CI) ^§^ |
| Sex (ref=m) | 0.41 (0.38, 0.44) | 0.34 (0.31, 0.38) |
| Ferritin, µg/L^‡^ | -0.04 (-0.07, -0.01) | 0.004 (-0.03, 0.04) |
| Hepcidin, nmol/l | -0.03 (-0.06, 0.002) | -0.20 (-0.03, 0.03) |
| sTfR, mg/l | -0.08 (-0.15, -0.01) | -0.12 (-0.20, -0.05) |
| TSAT, % | 0.11 (0.05, 0.17) | 0.23 (0.16, 0.29) |

^*^Model 3 was adjusted for age, BMI, smoking, alcohol use, total cholesterol, hs-CRP, eGFR, SBP, lipid-lowering drugs, antihypertensive drugs and T2D

^†^Model 4 was adjusted for age, BMI, smoking, alcohol use, total cholesterol, hs-CRP, eGFR, SBP, lipid-lowering drugs, antihypertensive drugs, T2D, and hemoglobin.

^‡^Analyses of iron biomarker were additionally adjusted for sex.

^§^Statistical test: multiple linear regression. All iron biomarkers and NT-proBNP were log transformed.

CI indicates confidence interval; sTfR, soluble transferrin receptor; TSAT, transferrin saturation; BMI, body mass index; hs-CRP, high-sensitivity c-reactive protein; eGFR, estimated glomerular filtration rate; SBP, systolic blood pressure; and T2D, type 2 diabetes mellitus.

Supplementary Table 2. Association of sex with iron biomarkers

| Iron biomarkers | Model 3^*^ | Model 4^†^ |
| --- | --- | --- |
|  | Beta (95% CI) ^‡^ | Beta (95% CI) ^‡^ |
| Ferritin, µg/L | -0.39 (-0.42, -0.36) | -0.24 (-0.27, -0.20) |
| Hepcidin, nmol/l | -0.21 (-0.24, -0.18) | -0.12 (-0.15, -0.08) |
| sTfR, mg/l | 0.02 (0.01, 0.03) | -0.01 (-0.03, 0.002) |
| TSAT, % | -0.07 (-0.08, -0.06) | 0.01 (-0.003, 0.03) |

^*^Model 3 was adjusted for age, BMI, smoking, alcohol use, total cholesterol, hs-CRP, eGFR, SBP, lipid-lowering drugs, antihypertensive drugs and T2D

^†^Model 4 was adjusted for age, BMI, smoking, alcohol use, total cholesterol, hs-CRP, eGFR, SBP, lipid-lowering drugs, antihypertensive drugs, T2D, and hemoglobin.

^‡^Statistical test: multiple linear regression. All iron biomarkers were log transformed.

CI indicates confidence interval; sTfR, soluble transferrin receptor; TSAT, transferrin saturation; BMI, body mass index; hs-CRP, high-sensitivity c-reactive protein; eGFR, estimated glomerular filtration rate; SBP, systolic blood pressure; and T2D, type 2 diabetes mellitus.

Reference category for sex is men.

Supplementary Table 3. Mediation analyses of iron biomarkers on the association between sex and NT-proBNP

| Iron biomarkers | Model 3^*^ | | |
| --- | --- | --- | --- |
|  | Effects^\|\|^ | Coefficient  (95% CI) | Mediated proportion  (%)^†^ |
| Ferritin, µg/L | Indirect  Total | 0.015 (0.003, 0.03)  0.40 (0.38, 0.43) | 4% ^‡^ |
| TSAT, % | Indirect  Total | -0.008 (-0.01, -0.003)  0.40 (0.38, 0.43) | 2% ^§^ |

In mediation analysis, only the iron biomarkers that were both associated with sex and NT-proBNP were evaluated.

^*^Model 3 was adjusted for age, BMI, smoking, alcohol use, total cholesterol, hs-CRP, eGFR, SBP, lipid-lowering drugs, antihypertensive drugs and T2D.

^†^Mediated proportion is calculated as the ratio of indirect effect to the total effect and is expressed in percentage.

^‡^Positive mediator effect; ^§^Negative mediator effect

^||^Statistical test: mediation analysis. All iron biomarkers and NT-proBNP were log transformed.

CI indicates confidence interval; TSAT, transferrin saturation; and BMI, body mass index; hs-CRP, high-sensitivity c-reactive protein; eGFR, estimated glomerular filtration rate; SBP, systolic blood pressure; and T2D, type 2 diabetes mellitus.

Supplementary Table 4. Association of iron status with NT-proBNP

| Iron status  (ref= Normal group) | Model 1^*^ | Model 2^†^ | Model 3^‡^ | Model 4^§^ |
| --- | --- | --- | --- | --- |
|  | Beta (95% CI) ^\|\|^ | | | |
| Absolute iron deficiency | 0.03 (-0.02, 0.07) | 0.02 (-0.02, 0.06) | 0.006 (-0.04, 0.05) | -0.05 (-0.10, -0.007) |
| Iron overload | 0.01 (-0.03, 0.05) | 0.02 (-0.02, 0.06) | 0.02 (-0.03, 0.06) | 0.02 (-0.02, 0.06) |

^*^Model 1 was adjusted for age and sex.

^†^Model 2 was adjusted for age, sex, BMI, smoking, and alcohol use.

^‡^Model 3 was adjusted for age, sex, BMI, smoking, alcohol use, total cholesterol, hs-CRP, eGFR, SBP, lipid-lowering drugs, antihypertensive drugs and T2D.

^§^Model 4 was adjusted for age, sex, BMI, smoking, alcohol use, total cholesterol, hs-CRP, eGFR, SBP, lipid-lowering drugs, antihypertensive drugs, T2D, and hemoglobin.

^||^Statistical test: multiple linear regression

CI indicates confidence interval; sTfR, soluble transferrin receptor; TSAT, transferrin saturation; BMI, body mass index; hs-CRP, high-sensitivity c-reactive protein; eGFR, estimated glomerular filtration rate; SBP, systolic blood pressure; and T2D, type 2 diabetes mellitus.

Supplementary Table 5. Association of sex with iron status

| Iron status  (ref= Normal group) | Model 1^*^ | Model 2^†^ | Model 3^‡^ | Model 4^§^ |
| --- | --- | --- | --- | --- |
|  | Odds Ratio (95% CI) ^\|\|^ | | | |
| Absolute iron deficiency | 2.78 (2.17, 3.56) | 2.5 (1.95, 3.23) | 2.48 (1.65, 3.73) | 0.52 (0.33, 0.82) |
| Iron overload | 0.20 (0.16, 0.30) | 0.20 (0.16, 0.26) | 0.22 (0.16, 0.30) | 0.28 (0.20, 0.41) |

^*^Model 1 was adjusted for age and sex.

^†^Model 2 was adjusted for age, sex, BMI, smoking, and alcohol use.

^‡^Model 3 was adjusted for age, sex, BMI, smoking, alcohol use, total cholesterol, hs-CRP, eGFR, SBP, lipid-lowering drugs, antihypertensive drugs and T2D.

^§^Model 4 was adjusted for age, sex, BMI, smoking, alcohol use, total cholesterol, hs-CRP, eGFR, SBP, lipid-lowering drugs, antihypertensive drugs, T2D, and hemoglobin.

^||^Statistical test: multiple logistic regression

CI indicates confidence interval; sTfR, soluble transferrin receptor; TSAT, transferrin saturation; BMI, body mass index; hs-CRP, high-sensitivity c-reactive protein; eGFR, estimated glomerular filtration rate; SBP, systolic blood pressure; and T2D, type 2 diabetes mellitus.

Reference category for sex is men.

Supplementary Table 6. Association of sex and iron biomarkers with NT-proBNP, after exclusion of participants who reported use of anti-hypertensive medications, lipid-lowering drugs or having diabetes

|  | Model 2* |
| --- | --- |
|  | Beta (95% CI) ^‡^ |
| Sex (ref=m) | 0.36 (0.33, 0.38) |
| Ferritin, µg/L^†^ | -0.04 (-0.07, -0.007) |
| Hepcidin, nmol/l | -0.03 (-0.06, 0.007) |
| sTfR, mg/l | -0.10 (-0.17, -0.02) |
| TSAT, % | 0.08 (0.02, 0.15) |

^*^Model 2 was adjusted for age, BMI, smoking, and alcohol use.

^†^Analyses of iron biomarker were additionally adjusted for sex.

^‡^Statistical test: multiple linear regression. All iron biomarkers and NT-proBNP were log transformed.

CI indicates confidence interval; sTfR, soluble transferrin receptor; TSAT, transferrin saturation; and BMI, body mass index.

Supplementary Table 7. Association of sex with iron biomarkers; after exclusion of participants who reported use of anti-hypertensive medications, lipid-lowering drugs or having diabetes

|  | Model 2* |
| --- | --- |
|  | Beta (95% CI) ^†^ |
| Ferritin, µg/L | -0.40 (-0.43, -0.38) |
| Hepcidin, nmol/l | -0.24 (-0.26, -0.22) |
| sTfR, mg/l | -0.002 (-0.01, 0.008) |
| TSAT, % | -0.08 (-0.09, -0.07) |

^*^Model 2 was adjusted for age, BMI, smoking, and alcohol use.

^†^Statistical test: multiple linear regression. All iron biomarkers were log transformed.

CI indicates confidence interval; sTfR, soluble transferrin receptor; TSAT, transferrin saturation; and BMI, body mass index.

Reference category for sex is men.

Supplementary Table 8. Mediation analyses of iron biomarkers on the association between sex and NT-proBNP, after exclusion of participants who reported use of anti-hypertensive medications, lipid-lowering drugs or having diabetes

|  | Model 2^*^ | | |
| --- | --- | --- | --- |
| Iron biomarkers | Effects^\|\|^ | Coefficient  (95% CI) | Mediated proportion  (%)^†^ |
| Ferritin, µg/L | Indirect  Total | 0.02 (0.002, 0.03)  0.36 (0.33, 0.38) | 5.5% ^‡^ |
| sTfR, mg/l | Indirect  Total | 0.0002 (-0.0008, 0.0013)  0.36 (0.33, 0.38) |  |
| TSAT, % | Indirect  Total | -0.007 (-0.01, -0.002)  0.36 (0.33, 0.38 | 1.9% ^§^ |

In mediation analysis, only the iron biomarkers that were both associated with sex and NT-proBNP were evaluated.

^*^Model 2 was adjusted for age, BMI, smoking, and alcohol use.

^†^Mediated proportion is calculated as the ratio of indirect effect to the total effect and is expressed in percentage.

^‡^Positive mediator effect; ^§^Negative mediator effect

^||^Statistical test: mediation analysis. All iron biomarkers and NT-proBNP were log transformed.

CI indicates confidence interval; sTfR, soluble transferrin receptor; TSAT, transferrin saturation; and BMI, body mass index.

Supplementary Table 9. Association of sex and iron biomarkers with NT-proBNP, based on median age, 51 years

| Model 2^*^ | Age group ≤51 years (n=2681) | Age group >51 years (n=2662) |
| --- | --- | --- |
|  | Beta (95% CI) ^‡^ | Beta (95% CI) |
| Sex (ref=m) | 0.45 (0.41, 0.48) | 0.25 (0.21, 0.28) |
| Ferritin, µg/L^†^ | 0.02 (-0.05, 0.08) | -0.02 (-0.06, 0.02) |
| Hepcidin, nmol/l | -0.004 (-0.07, 0.06) | -0.004 (-0.05, 0.04) |
| sTfR, mg/l | -0.12 (-0.26, 0.02) | -0.12 (-0.22, 0.03) |
| TSAT, % | 0.12 (0.004, 0.23) | 0.07 (-0.01, 0.15) |

^*^Model 2 was adjusted for age, BMI, smoking, and alcohol use.

^†^Analyses of iron biomarker were additionally adjusted for sex.

^‡^ Statistical test: multiple linear regression. All iron biomarkers and NT-proBNP were log transformed.

CI indicates confidence interval; sTfR, soluble transferrin receptor; TSAT, transferrin saturation; and BMI, body mass index.

Supplementary Table 10. Association of sex with iron biomarkers, based on median age, 51 years

| Model 2^*^ | Age group ≤51 years (n=2681) | Age group >51 years (n=2662) |
| --- | --- | --- |
|  | Beta (95% CI) ^†^ | Beta (95% CI) |
| Ferritin, µg/L | -0.56 (-0.60, -0.52) | -0.40 (-0.42, -0.36) |
| Hepcidin, nmol/l | -0.41 (-0.45, -0.36) | -0.23 (-0.26, -0.20) |
| sTfR, mg/l | 0.000 (-0.02, 0.02) | -0.004 (-0.01, 0.008) |
| TSAT, % | -0.10 (-0.12, -0.07) | -0.07 (-0.08, -0.06) |

^*^Model 2 was adjusted for age, BMI, smoking, and alcohol use.

^†^ Statistical test: multiple linear regression. All iron biomarkers were log transformed.

CI indicates confidence interval; sTfR, soluble transferrin receptor; TSAT, transferrin saturation; and BMI, body mass index.

Supplementary Table 11. Mediation analyses of iron biomarkers on the association between sex and NT-proBNP, based on median age, 51 years

| Model 2* | Age group ≤51 years (n=2681) | | |
| --- | --- | --- | --- |
| Iron biomarkers | Effects^§^ | Coefficient  (95% CI) | Mediated proportion  (%)^†^ |
| TSAT, % | Indirect  Total | -0.01 (-0.02, -0.007)  0.47 (0.42, 0.51) | 2.1% ^‡^ |

In mediation analysis, only the iron biomarkers that were both associated with sex and NT-proBNP were evaluated.

^*^Model 2 was adjusted for age, BMI, smoking, and alcohol use.

^†^Mediated proportion is calculated as the ratio of indirect effect to the total effect and is expressed in percentage.

^‡^Negative mediator effect

^§^Statistical test: mediation analysis. All iron biomarkers and NT-proBNP were log transformed.

CI indicates confidence interval; TSAT, transferrin saturation; and BMI, body mass index.
